# Longevity, aging and rapamycin

**DOI:** 10.1007/s00018-014-1677-1

**Published:** 2014-07-12

**Authors:** Dan Ehninger, Frauke Neff, Kan Xie

**Affiliations:** 1 German Center for Neurodegenerative Diseases (DZNE), Ludwig-Erhard-Allee 2, 53175 Bonn, Germany; 2 Institute of Pathology, Helmholtz Zentrum München, Ingolstädter Landstraße 1, 85764 Neuherberg, Germany; 3 German Mouse Clinic, Institute of Experimental Genetics, Helmholtz Zentrum München, Ingolstädter Landstraße 1, 85764 Neuherberg, Germany

**Keywords:** mTOR, Mammalian target of rapamycin, Longevity, Lifespan, Aging, Rapamycin, Mice, Mammals, Disease, Treatment, Drug, Prevention, Mechanism, Neurodegeneration, Cardiovascular disease, Cancer, Anti-aging

## Abstract

The federal drug administration (FDA)-approved compound rapamycin was the first pharmacological agent shown to extend maximal lifespan in both genders in a mammalian species. A major question then is whether the drug slows mammalian aging or if it has isolated effects on longevity by suppressing cancers, the main cause of death in many mouse strains. Here, we review what is currently known about the effects that pharmacological or genetic mammalian target of rapamycin (mTOR) inhibition have on mammalian aging and longevity. Currently available evidence seems to best fit a model, wherein rapamycin extends lifespan by suppressing cancers. In addition the drug has symptomatic effects on some aging traits, such as age-related cognitive impairments.

## Introduction

Aging is a major risk factor for a range of diseases in numerous organ systems, including cardiovascular disease, neurodegenerative diseases, cancers, diabetes mellitus type II, osteoporosis etc. Interventions that target the molecular processes underlying aging could therefore provide novel entry points for the development of innovative preventatives and/or therapeutics for a range of age-related diseases [1, 2].

Much research over the past ~2 decades has focused on the identification of genetic mutations that extend lifespan in invertebrate model organisms. Pathways involved in the control of cell growth and metabolism have emerged as important players of lifespan regulation [3, 4]. Mammalian target of rapamycin (mTOR) is a kinase at a key signalling node that integrates information regarding extracellular growth factor stimulation, nutrient availability and energy supplies [5]. A number of studies in yeast [6], worms [7, 8] and flies [9] have initially implicated mTOR in lifespan control.

The recent discovery that the mTOR inhibitor rapamycin extends mammalian lifespan [10] has created much excitement because it represented the first demonstration of pharmacological extension of maximal lifespan in a mammalian species. Rapamycin’s mammalian longevity effects have since then been confirmed by a number of additional studies [11–15]. Rapamycin is FDA-approved and considerable experience exists with the clinical application of this drug: Rapamycin and derivates of this compound are used clinically to prevent organ rejection after kidney transplantation and also to prevent occlusion of cardiac stents. mTOR inhibitors are also clinically tested for the treatment of cancers and neurogenetic disorders, such as tuberous sclerosis. Translational studies that assess rapamycin’s effects on human aging and age-related disease are therefore thought to be within reach and have actually been initiated at some sites (ClinicalTrials.gov: NCT01649960; [16]).

Although mTOR inhibition has been clearly shown to extend murine lifespan [10–15, 17] and also to have beneficial effects on a set of murine aging traits [11–14, 17–22], there is currently little evidence available to support the notion that mTOR inhibitors slow the rate of mammalian aging. In this article, we review what is currently known about the effects that pharmacological or genetic mTOR inhibition have on mammalian aging and longevity.

## Rapamycin and mTOR signaling

**Fig. 1:**
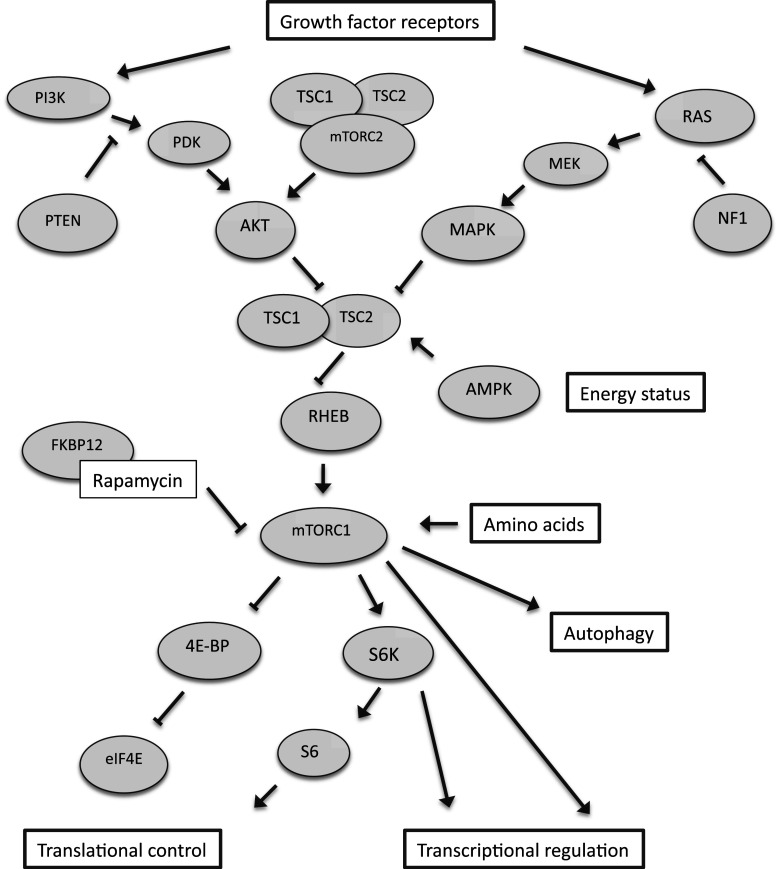
Schematic illustration of mTOR-related cell signaling. *AKT* serine/threonine protein kinase, *AMPK* AMP-activated protein kinase, *FKBP12* 12 kDa FK506-binding protein, *eIF4E* eukaryotic translation initiation factor 4E, *4E-BP* eIF4E binding protein, *MAPK* mitogen-activated protein kinase, *MEK* mitogen-activated protein kinase kinase, *mTORC1* mammalian target of rapamycin complex 1, *mTORC2* mammalian target of rapamycin complex 2, *NF1* neurofibromin, *PDK* phosphoinositide-dependent protein kinase, *PI3* *K* phosphoinositide-3-kinase, *PTEN* phosphatase and tensin homolog, *Ras* G protein Ras, *Rheb* Ras homologue enriched in brain, *S6* *K* S6 kinase, *S6* ribosomal protein S6, *TSC1* tuberous sclerosis protein 1, *TSC2* tuberous sclerosis protein 2. Modified with permission from [79]

mTOR occurs in two distinct protein complexes, namely mTORC1 and mTORC2 [23] (see Fig. 1; for abbreviations, see Fig. 1 legend). The mTORC1 protein complex includes raptor (regulatory-associated protein of mTOR) and mLST8 in addition to mTOR. Upstream regulatory signaling inputs that converge onto mTORC1-related cell signaling include the PI3K/AKT [24, 25] and Ras/MAPK pathways [26, 27], as well as AMPK signaling [28]. mTORC1 is therefore well-positioned to coordinate cellular growth-related processes and integrate them with the availability of nutrients, energy and the appropriate stimulation of growth factor receptors.

mTORC1 plays an important role in the regulation of a range of cellular processes, including de novo protein synthesis [5, 29, 30]: mTORC1 stimulates the translation of mRNAs with a highly structured 5′ untranslated region (5′UTR) by phosphorylating 4E-BPs, thereby derepressing eIF4E and, consecutively, promoting translational initiation. Additionally, mTORC1 controls protein synthesis via the p70S6 kinase/ribosomal protein S6 pathway, which stimulates the translation of mRNAs with a 5′ terminal oligopyrimidine tract (5′TOP), many of which encode for components of the translational machinery (e.g., ribosomal subunits, translation factors etc.). Experiments in *C. elegans* showed that a number of different genetic manipulations affecting the protein synthesis machinery (such as genetic deletion or siRNA-mediated knock-down of ribosomal subunits and translation factors, respectively) are associated with extended lifespan [31–33], indicating that altered translational rates could contribute to longevity effects of mTOR inhibition in this organism. In mice, lifespan extension was observed in female mice with a homozygous mutation in ribosomal S6 protein kinase 1 (S6K1) [34]. Whether mammalian aging rates are slowed by translational modulation remains unknown.

Another important cellular process regulated by mTORC1 signaling is autophagy. Autophagy, a process by which the cell recycles macromolecules and organelles, allows for the removal of damaged cellular constituents and enables the cell to mobilize substrate under nutrient-poor conditions. mTORC1 regulates autophagy by phosphorylating and inhibiting the autophagy-initiating kinase Ulk1 [35]. In *C. elegans*, autophagy has been reported to be required for the lifespan extension caused by genetic mTOR inhibition [36]. Available evidence in mice indicates that aspects of liver aging (age-related histopathological and functional liver changes) are improved when an inducible genetic system is employed to increase autophagy levels in aged mice [37]. Whether murine lifespan extension, caused by rapamycin, is dependent on autophagy effects has not been addressed to date.

In the context of the mTORC2 protein complex, mTOR is associated with rictor (rapamycin-insensitive companion of mTOR), GβL and mSIN1 (mammalian stress-activated protein kinase interacting protein). mTORC2 is involved in regulating the activity of AKT [38]. In contrast to mTORC1, which is inhibited by rapamycin, mTORC2 is rapamycin-insensitive. Prolonged rapamycin treatment, however, inhibits mTORC2 indirectly [39].

The mechanisms by which rapamycin inhibits mTOR are not fully understood; it is, however, established that rapamycin associates with FKBP12 to bind to mTOR’s FRB (FKBP12-rapamycin-binding) domain. Binding of the rapamycin-FKBP12 complex to mTOR may destabilize the mTORC1 complex [40] and, in addition, interfere with the activation of mTOR by phosphatidic acid [41]. Novel mTOR inhibitors are available that either inhibit mTOR by interfering with mTORC1 complex formation (FKBP12-dependent or FKBP12-independent) or by directly inhibiting mTOR’s catalytic domain [42].

## Rapamycin extends lifespan in mice

In their important 2009 paper, the NIA’s intervention testing program (ITP) identified rapamycin as the first pharmacological agent to extend maximal lifespan in a mammalian species, with effects in both males and females [10] (Table 1). Rapamycin was encapsulated (to increase bioavailablity of the drug) and delivered via the mouse chow at a concentration of 14 parts per million (ppm). In this study, treatment was started at two different ages: 270 days and 600 days, respectively. Both treatments were found to effectively extend maximal lifespan in a genetically heterogeneous stock of mice (UM-HET3 mice) [10] and the effect size did not differ in any obvious way between the earlier-onset and the later-onset treatment [11]. More recently, the longevity effect of two additional doses of rapamycin has been examined [15]: in this follow-up study, the ITP assessed rapamycin orally administered to initially 9 months old UM-HET3 mice at the concentrations of 4.7, 14 or 42 ppm. All three doses extended maximal and median lifespan in females and the two higher doses (14, 42 ppm) caused significant lifespan extension in males (also both maximal and median lifespan extension) [15]. Lifespan-extending effects were larger in females than in males, most likely because of higher rapamycin blood concentrations found in females at a given rapamycin chow concentration ([15]; although these gender differences in rapamycin blood concentrations were not observed in all studies [10]). It remains to be determined whether these possible gender differences in rapamycin blood levels are related to sex differences in food intake, resorption of the drug, tissue distribution, metabolism of the drug or a combination of these factors. Rapamycin has also been shown to extend lifespan in mice of other genetic backgrounds, such as C57BL/6 [12, 13] and 129/Sv [14] (Table 1). Additionally, there is also genetic evidence implicating loss of mTOR function in murine lifespan extension: animals homozygous for a hypomorphic *mTOR* mutation (decreasing mTOR expression to 25 % of wildtype levels) show a lifespan extension that is also seen across both males and females [17] (Table 1).

**Table 1:** Mammalian longevity studies using rapamycin or genetic mTOR inhibition

Intervention	Strain, sex	Lifespan effects	Cause of death analysis	References
Oral rapamycin (encapsulated, 14 ppm) initiated at 270 days or 600 days of age	Male and female UM-HET3 mice	Extension of median and maximal lifespan in both genders	Both treated animals and controls die due to cancers in >80 % of cases, but treated animals do so later in life	[10, 11]
Oral rapamycin (encapsulated; 4.7, 14 or 42 ppm) initiated at 9 months of age	Male and female UM-HET3 mice	All three doses extended median and maximal lifespan in females; the two higher doses (i.e., 14 and 42 ppm) extended median and maximal lifespan in males	Not performed	[15]
Rapamycin was injected i.p. at 4 mg/kg once every other day for 6 weeks, starting at 22–24 months	Male C57BL/6 mice	Survival higher in rapamycin group than in control group (follow up for 30 weeks after first injection)	Not performed	[12]
Oral rapamycin (encapsulated, 14 ppm) for approx. 1 year starting at 4 months, 13 months or 25 months; animals were then subjected to a comprehensive analysis of aging phenotypes	Male C57BL/6J Rj mice	Rapamycin extended lifespan (follow up until completion of phenotypic aging analysis was completed)	Not performed	[13]
Rapamycin was injected s.c. at 1.5 mg/kg 3 times a week for a period of 2 weeks followed by 2 weeks without rapamycin. Treatment started at 2 months and continued to natural death of the animals	Female 129/Sv mice	Rapamycin specifically extended lifespan in tumor-bearing animals, but had no significant effect on longevity in tumor-free animals	Necropsies were performed to assess the contribution of neoplasias to death of the animals; in the control group >70 % of animals showed tumors on necropsy; in the rapamycin group approx. 30 % showed tumors on necropsy	[14]
Hypomorphic *mTOR* allele (*mTOR* ^∆/∆^);* mTOR* ^∆/∆^ mice were compared to wildtype littermate controls	Male and female mice on a mixed 129S1 and C57BL/6Ncr background	Extension of median survival in both male and female *mTOR* ^∆/∆^ mice; also, probable extension of maximal lifespan (but low sample size)	Necropsies showed reduced incidence of malignant tumors, but higher rates of infections in *mTOR* ^∆/∆^ mice	[17]

## Rapamycin longevity studies: why do treated animals live longer?

As mentioned above, the rapamycin longevity studies in mice published to date examined several genetic backgrounds, namely inbred C57BL/6 backgrounds [12, 13], 129/Sv [14] and the genetically heterogeneous UM-HET3 stock of animals (the stock used by the NIA’s Intervention Testing Program) [10, 11, 15] (see Table 1).

In all these backgrounds and across sexes, neoplastic lesions represent a major cause of death. For example, approx. 70 % of C57BL/6 animals naturally die due to neoplastic disease with lymphomas and hematopoietic neoplasms representing the leading causes of death [43–45]. Similarly, in UM-HET3 mice, neoplastic lesions are the natural cause of death in >80 % of cases [11, 46]. Lymphomas and hematopoietic tumors also represent the most common neoplastic lesions that naturally limit life in UM-HET3 mice [11, 46]. Any intervention extending lifespan in these strains is, therefore, expected to do so primarily by counteracting these common life-limiting neoplastic pathologies.

Lifespan extension via inhibition of carcinogenesis is indeed a plausible scenario for rapamycin-mediated longevity effects because rapamycin has well-known anti-neoplastic properties, including inhibitory effects on de novo cancer formation, as well as suppression of established tumors via inhibition of cancer growth, promotion of apoptosis of neoplastic cells and/or a modification of the host response to the tumor (for example, inhibiting angiogenesis) [47–54]. In line with this, rapamycin was found to suppress cancers and extend life in a range of genetic early-onset cancer models, such as p53 mutant mice, Apc mutant animals, Rb mutant mice and HER-2/neu transgenic mice [55–58], strongly implicating direct anti-cancer action in the longevity effects seen in these studies.

Detailed cause-of-death analyses in rapamycin-treated UM-HET3 mice and controls indicated that both groups die primarily (i.e., in >80 % of cases) due to cancers, but rapamycin-treated animals do so later in life than controls [11], indicating that rapamycin postpones lethal neoplastic disease in treated animals. In the context of this study, it was not possible to determine if rapamycin also extends lifespan in those animals that die due to non-neoplastic disease because non-neoplastic disease accounted only for a small fraction (approx. 10 %) of deaths in UM-HET3 mice [11].

An analysis of rapamycin’s lifespan effects in aging 129/Sv female mice showed a clear lifespan extension and a reduced tumor burden in treated animals [14]. Further analyses in 129/Sv mice indicated that rapamycin-mediated lifespan extension is only seen in animals that eventually die due to neoplastic disease; in contrast, rapamycin did not significantly extend lifespan in those animals that die due to non-neoplastic disease [14], supporting the notion that rapamycin extended lifespan by specifically inhibiting neoplasia-related lethality (without effects on lethality related to non-neoplastic disease).

Similarly, a study analyzing lifespan and end-of-life pathology in hypomorphic *mTOR* mutant mice reported a clear reduction of malignant tumors in the mutants, while infections were more common in these animals [17]. Reduced numbers of precancerous lesions and cancers were also found in rapamycin-treated aging C57BL/6J mice [13].

Together, the data available (discussed above) indicate that rapamycin primarily extends mammalian lifespan by inhibiting lethal neoplastic disease. It will be important to assess rapamycin’s effects on additional mouse strains and/or other mammalian species that show a broader spectrum of additional non-neoplastic pathology as contributory factors to death.

## Aging research: from lifespan to healthspan measures

Studies over the past ~20 years have identified a large number of genetic manipulations that extend life in invertebrate model organisms, such as *Caenorhabditis elegans* and *Drosophila melanogaster*. Some of the pathways identified were also shown to be involved in the regulation of mammalian lifespan (using mice) [59], although mechanisms of lifespan extension in these different species are likely very different (at least the proximal mechanisms; see discussion above).

Lifespan extension does not necessarily indicate effects on aging. With regards to lifespan-extending interventions in mice two general scenarios are possible: (a) interventions may have isolated effects on lifespan by inhibiting specific life-limiting pathology, such as cancers, without broadly modulating aging traits; (b) lifespan extension occurs by inhibition of life-limiting pathologies, such as cancers, and this effect represents one aspect of a more general effect that the respective intervention has on aging.

During the course of aging most mammalian tissues and organ systems undergo characteristic molecular, structural and functional alterations. To assess whether a pharmacological or genetic intervention slows the rate of mammalian aging it is necessary to examine its effects on a broad range of mammalian aging phenotypes in different cell types, tissues and organ systems. Comprehensive analyses of aging traits may then identify aging traits that are ameliorated by specific genetic or pharmacological interventions.

## Modulation of aging traits: slowing aging vs. symptomatic effects on aging traits

A given intervention that ameliorates aging traits could in principle do so via one of two ways: (a) causally, by slowing the rate of aging, that is by slowing the process(es) that underlie the age-dependent development of the respective aging trait(s); (b) symptomatically, i.e., by modifying the respective traits in ways that are mechanistically distinct from the processes that underlie the aging traits’ development.

Experimentally, one can reveal the symptomatic nature of the modulation of an aging trait by administering the intervention well before the respective aging trait has developed. C57BL/6J mice, for example, develop aging-associated deficits in spatial reference memory tasks, such as the Morris water maze, at some point during their second year of life or thereafter [60, 61]. If a one-month treatment with a drug (e.g., a stimulant psychoactive compound) enhances spatial memory in 4 months old C57BL/6J mice, then it is clear that this drug improves memory by tapping into mechanisms that are distinct from those that underlie aging-associated cognitive decline. If it is then found that a long-term treatment with this same drug enhances memory in 2 year old mice, the most parsimonious explanation of this set of findings is that the drug affects age-dependent memory impairments symptomatically (i.e., without affecting the rate of cognitive aging).

**Fig. 2:**
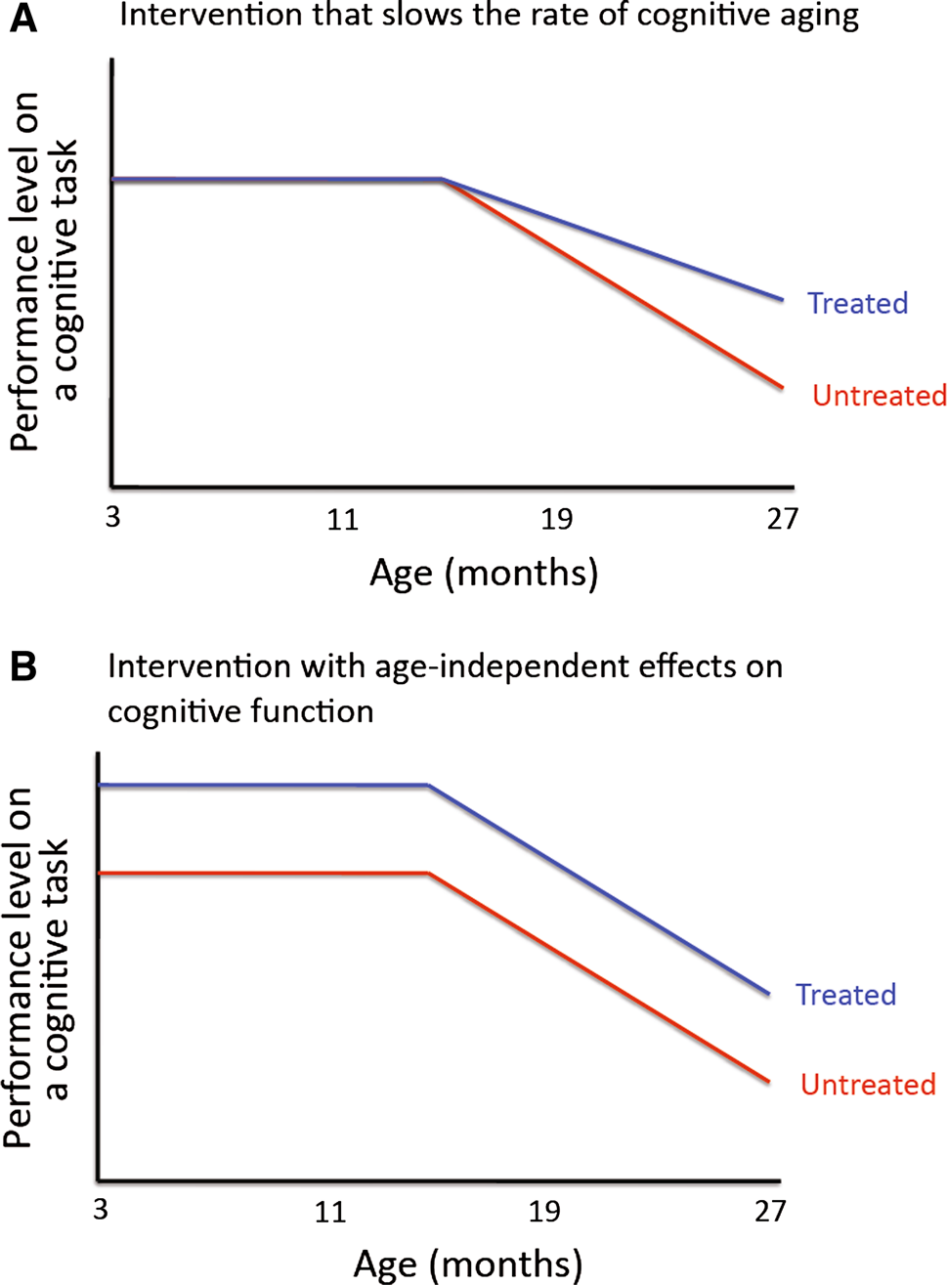
Causal versus symptomatic effects on aging traits. A putative anti-aging intervention could in principle exert its effects on a given aging trait causally, by slowing the rate of aging (**a**), or symptomatically via aging-independent effects (**b**). **a** and **b** show examples of age-related decline of performance on a cognitive task that progressively starts in the second year of life of the animal. A treatment (given throughout life) that slows the rate of cognitive aging would be expected to specifically affect performance on the task once age-related decline has started (**a**). A treatment (given throughout life) that symptomatically improves age-related cognitive decline would be expected to improve performance at all ages (**b**)

This principle is illustrated in Fig. 2: interventions that slow the rate of aging are expected to specifically interfere with the age-related change of specific traits (e.g., age-related decline in performance on certain cognitive tasks), but do not have the same effects on young organisms that did not yet express the respective aging trait (i.e., no enhancement of cognitive performance in young organisms that did not yet develop aging-associated cognitive impairments). Other interventions, in contrast, may have symptomatic effects on an aging trait without slowing the rate of aging itself: These interventions would be expected to influence the respective trait in young, as well as in old individuals.

## Rapamycin effects on aging traits in mice

Several studies have assessed rapamycin’s effects on aging traits and they cover numerous aging phenotypes in different cell types, tissues and organ systems [11–14, 18–22]. Most of these studies have employed the ITP’s original rapamycin treatment regimen (i.e., encapsulated rapamycin administered via the mouse chow at a concentration of 14 ppm) that has been repeatedly shown to extend maximal and median lifespan in both males and females. Rapamycin shows extensive tissue distribution [13, 22, 62] and is therefore available for mTOR inhibition in numerous organs. The ITP’s rapamycin treatment regimen was shown to inhibit phosphorylation of ribosomal protein S6 at the mTOR-sensitive sites Ser240/244 in a number of tissues of both male and female mice, including heart, liver, kidney and fat [10, 22]. Similarly, this treatment protocol was also found to increase the LC3II/LC3I ratio in the tissues mentioned above [22], which is indicative of enhanced autophagy in treated animals. The by far most comprehensive assessment of rapamycin’s effects on murine aging was performed by Neff et al. [13] in a study that covered >150 aging traits across >25 tissues. The Neff et al. study is the only published report that took aging-independent drug effects into consideration when assessing rapamycin’s effects on aging traits. In the sections below, we review the current state of knowledge regarding rapamycin effects and effects of genetic mTOR inhibition on murine aging traits.

## Neurological findings

Aging is associated with a number of neurobehavioral and neurological changes, such as cognitive decline, alterations in motor coordination, balance and reduced muscle strength [13, 60, 61, 63]. Rapamycin effects on neurological aging traits were assessed in several studies [11, 13, 18–22] and the observations are summarized below (see also Table 2).

**Table 2:** Neurological findings

Spontaneous locomotor activity						
Assay	Aging phenotype	Intervention	Strain, sex	Treatment effects in aged mice	Treatment effects in young mice	References
Assessment of exploratory activity in a novel environment (open field, 20 min)	Reduced exploratory activity	Aging cohorts: oral rapamycin (14 ppm) for approx. 1 year starting at 4 months, 13 months or 25 months; young animals: oral rapamycin (14 ppm) for 3 months starting at 12 weeks of age	Male C57BL/6J Rj	Increase in exploratory activity	Increase in exploratory activity	[13]
Assessment of spontaneous in-cage motor activity (for 50 h); baseline assessment at 7 months of age; animals reexamined at 18 months of age	Reduced motor activity	Oral rapamycin (14 ppm) initiated at 9 months of age	Male UM-HET3 mice	Amelioration of age-dependent reduction in motor activity (in 2 out of 3 cohorts)	Not examined	[11]
Assessment of spontaneous in-cage motor activity (for 50 h); baseline assessment at 7 months of age; animals reexamined at 18 months of age	Reduced motor activity	Oral rapamycin (4.7, 14 or 42 ppm) initiated at 9 months of age	Male and female UM-HET3 mice	Amelioration of age-dependent reduction in motor activity (significant in males given 14 ppm and females given 42 ppm)	Not examined	[18]
Voluntary wheel running (assessed for 48 h)	Not examined	Oral rapamycin (14 ppm) for 3 months started at 24 months of age	Female C57BL/6J	Increased voluntary wheel running	Not examined	[19]

Motor coordination and balance						
Assay	Aging phenotype	Intervention	Strain, sex	Treatment effects in aged mice	Treatment effects in young mice	References
Accelerating rotarod	Reduced latencies to fall	Oral rapamycin (14 ppm) for approx. 1 year starting at 4, 13 or 25 months	Male C57BL/6J Rj	No effect	Not examined	[13]
Accelerating rotarod (shown are results of a 5th session after 4 sessions of training; rotarod assessment at 25 and 31 months of age)	No decline in latencies to fall between 25 and 31 months	Oral rapamycin (14 ppm) starting at 19 months of age	Male and female C57BL/6Nia mice	Increased latencies to fall at 31 months	Not examined	[22]
Accelerating rotarod		Hypomorphic *mTOR* allele (*mTOR* ^∆/∆^);* mTOR* ^∆/∆^ mice were compared to wildtype littermate controls; young animals were 3–6 months old; aged animals were 17–27 months old	Mixed 129S1 and C57BL/6Ncr background; male mice	Increased latencies to fall in aged *mTOR* ^∆/∆^ mice (caveat: small sample size with only 4–6 mice per group; body weight differences between mutants and controls not accounted for)	No effect	[17]
Stride width analysis		Hypomorphic *mTOR* allele (*mTOR* ^∆/∆^); *mTOR* ^∆/∆^ mice were compared to wildtype littermate controls; young animals were 3–6 months old; aged animals were 17–27 months old	Mixed 129S1 and C57BL/6Ncr background; female or mixed male/female groups of mice	Reduced stride width variance in aged *mTOR* ^∆/∆^ mice (caveat: small sample size with only 6 mice per group in all but 1 group)	No effect	[17]
Automated gait analysis on TreadScan apparatus (40 gait parameters were analyzed); assessment at 25, 30 and 32 months		Oral rapamycin (14 ppm) starting at 19 months of age	Male and female C57BL/6Nia mice	Increased stride length	Not examined	[22]

Muscle strength						
Assay	Aging phenotype	Intervention	Strain, sex	Treatment effects in aged mice	Treatment effects in young mice	References
Grip strength analysis	Reduced grip strength	Oral rapamycin (14 ppm) for approx. 1 year starting at 4, 13 or 25 months	Male C57BL/6J Rj	No effect	Not examined	[13]
Grip strength analysis	No difference in grip strength between 25 and 31 months	Oral rapamycin (14 ppm) starting at 19 months of age	Male and female C57BL/6Nia mice	No effect	Not examined	[22]
Grip strength analysis	Reduced grip strength	Hypomorphic *mTOR* allele (*mTOR* ^∆/∆^);* mTOR* ^∆/∆^ mice were compared to wildtype littermate controls; young animals were 3–6 months old; aged animals were 17–27 months old	Mixed 129S1 and C57BL/6Ncr background; female or mixed male/female groups of mice	Increased grip strength in aged *mTOR* ^∆/∆^ mice (caveat: small sample size with only 4–6 mice per group in 3 out of 4 groups)	No effect	[17]

Nociception						
Assay	Aging phenotype	Intervention	Strain, sex	Treatment effects in aged mice	Treatment effects in young mice	Reference
Hot plate test	Increased latencies to reaction	Oral rapamycin (14 ppm) for approx. 1 year starting at 4 months	Male C57BL/6J Rj	No effect	Not examined	[13]

Learning and memory						
Assay	Aging phenotype	Intervention	Strain, sex	Treatment effects in aged mice	Treatment effects in young mice	References
Morris water maze	Increased escape latencies during training; no age effect on probe trial measures (comparing 11 months vs. 20 months old animals)	Aging cohorts: oral rapamycin (14 ppm) for approx. 7 months starting at 4 or 13 months of age; young animals: oral rapamycin (14 ppm) for 6 weeks starting at 6 months of age	Male C57BL/6J Rj	Improved probe trial performance and decreased latencies during training	Improved probe trial performance and decreased latencies during training	[13]
Morris water maze	Increased escape latencies during training; reduced target crossings and target quadrant occupancy during a probe trial	Oral rapamycin (14 ppm) for approx. 16 months starting at 2 months of age	C57BL6/129svj mice; sex not reported	Improved probe trial performance and decreased latencies during training	Not examined	[20]
Morris water maze	Not examined	Oral rapamycin (14 ppm) for 4 months starting at 4 months of age	Male C57BL/6J	Not examined	Improved probe trial performance and decreased latencies during training	[21]
Barnes maze	Increased escape latencies	Hypomorphic *mTOR* allele (*mTOR* ^∆/∆^); *mTOR* ^∆/∆^ mice were compared to wildtype littermate controls; young animals were 3–6 months old; aged animals were 17–27 months old	Mixed 129S1 and C57BL/6Ncr background; female groups of mice	Decreased escape latencies in the aged mutants (caveat: small sample size with some groups having only 6 mice)	No significant difference, but trend in the same direction (possible floor effect)	[17]
Object place recognition	Reduced exploration of novel object during test	Oral rapamycin (14 ppm) for approx. 7 months starting at 4 or 13 months of age	Male C57BL/6J Rj	No effect	Not examined	[13]
Context fear conditioning	No significant effect of age observed (comparing 15, 24 and 33 months old mice)	Oral rapamycin (14 ppm) for approx. 1 year starting at 4, 13 or 20–22 months of age	Male C57BL/6J Rj	Enhanced fear conditioning (lower activity suppression ratios)	Not examined	[13]

Aging in mice is typically associated with reduced levels of exploration and locomotor activity [11, 13, 18]. One robust finding, seen across a number of studies, is rapamycin’s stimulatory effect on locomotor activity in treated animals [11, 13, 18, 19]. Miller et al. [11] and Wilkinson et al. [18] measured spontaneous motor activity of animals in their cages for an extended time period (50 h). They examined animals at two time points, first at 7 months of age and, in those that survived, again at 18 months of age. Rapamycin or vehicle control treatment was initiated at 9 months of age. Their findings indicated that the age-dependent reduction of motor activity is ameliorated in animals aged on rapamycin [11, 18]. Stimulatory rapamycin effects on motor activity were also noted in another study [19], in the context of which voluntary wheel running was measured in aged animals subjected to oral rapamycin or vehicle control chow for 3 months starting at the age of 24 months.

Neff et al. [13] examined exploratory activity in an open field assay in three cohorts of animals that were aged on rapamycin or vehicle control for approx. 1 year, starting at either 4, 13 or 25 months. Exploratory activity levels were substantially reduced in aged mice relative to young controls and rapamycin treatment significantly elevated exploratory activity in aged animals [13]. The same rapamycin treatment regimen, however, also increased exploratory activity in young mice (3 months old animals that were treated for 12 weeks before testing commenced) [13], indicating that these drug effects were probably not related to a modulation of aging, but that they rather represented aging-independent drug effects on locomotor activity.

Collectively, the studies mentioned above demonstrate stimulatory effects of rapamycin on locomotor activity that are observable across genetic backgrounds (UM-HET3, C57BL/6 J) and affect males, as well as females. Relatively shorter-term (i.e., 3 months) rapamycin treatment is sufficient to increase motor activity and this is the case also for young animals (treated from 3 to 6 months of age), indicating that these rapamycin effects are age-independent.

Another robust neurobehavioral finding in animals subjected to the ITP rapamycin protocol is an enhanced performance on a number of different learning and memory tasks [13, 20, 21] (Table 2). Reduced escape latencies and improved probe trial performance in the Morris water maze in rapamycin-treated animals was observed by a number of laboratories and across different genetic backgrounds (C57BL6/J, mixed C57BL6/129svj) [13, 20, 21]. Rapamycin treatment, however, improved learning and memory in the Morris water maze not only in animals that were aged on the drug, but also in young adult mice (treatment starting at 6 months and lasting for 6 weeks) [13]––i.e., well before the onset of age-related cognitive decline. The ITP rapamycin protocol has therefore clear enhancing effects on learning and memory that can be employed to symptomatically improve cognition in aged mice.

Additional neurological studies in rapamycin-treated aged animals included assessments of motor coordination and balance [13, 22], gait [22], muscle strength [13, 22], nociception [13] and histopathological assessments of aging traits in muscle and brain [13]. The ITP rapamycin regimen applied for 1 year did not measurably improve performance on an accelerating rotarod in cohorts of 16 months and 25 months old animals, respectively (treatment commenced at 4 months and 13 months, respectively) [13], indicating limited effects of treatment on this aging trait. Another study employed a longitudinal assessment of rotarod performance at 25 months and 31 months in mice subjected to treatment that was started at 19 months [22]. Although there was no decline in rotarod performance between 25 and 31 months in the controls, rapamycin treatment was reported to improve performance at the age of 31 months [22].

Rotarod performance was also examined in hypomorphic *mTOR* mutant mice (*mTOR* ^∆/∆^ mice; [17]). The study showed the expected age effect on rotarod performance (old animals performing worse then young animals; for details, see Table 2). Aged *mTOR* ^∆/∆^ mice performed better than aged wildtype controls, while there was no genotype effect (*mTOR* ^∆/∆^ mice vs. WT) in the young cohort of animals. This may indicate that the *mTOR* ^∆/∆^ mutation has age-dependent effects on motor coordination and balance. Limitations associated with this experiment, however, were small sample size (only 4–6 animals per group) and the fact that body weight differences between mutants and controls (*mTOR* ^∆/∆^ mice are smaller and weigh less than their wildtype counterparts [17]) were not accounted for in the analysis (body weight and latencies to fall are inversely correlated; [64]).

The ITP rapamycin protocol did not measurably affect age-related impairments in muscle strength, as assessed in the grip strength test [13, 22]. Additionally, this treatment regimen did not modify the aging-associated decline in cross-sectional muscle fiber area in the quadriceps femoris muscle [13]. Wu et al. [17] reported increased muscle strength in aged *mTOR* ^∆/∆^ mice relative to aged wildtype controls, while the *mTOR* ^∆/∆^ genotype did not affect grip strength in young animals. While these data may suggest that the hypomorphic *mTOR* mutation slows the development of aging-associated grip strength impairments, it will be important to corroborate this observation using larger groups of animals (the groups contained only 4–6 animals in most groups).

In sum, oral rapamycin has stimulatory effects on locomotor behavior [11, 13, 18, 19] and improves learning and memory [13, 20, 21]. These are robust findings seen across mouse strains and genders. Because rapamycin has similar effects in young animals and aging cohorts [13], it is the most parsimonious explanation of the data that these rapamycin effects are not related to a modulation of aging. The oral rapamycin ITP protocol had limited effects on motor coordination and balance [13, 22], muscle strength [13, 22], sarcopenia [13] and age-related nociceptive dysfunction [13]. Preliminary evidence suggests that genetic mTOR inhibition in hypomorphic *mTOR* mutant mice may result in preserved motor coordination and muscle strength in aged animals [17].

## Ophthalmological findings

A common aging-associated pathology affecting the anterior part of the eye is cataract formation [65]. Two studies published to date assessed rapamycin effects on age-related lens density alterations [13, 18] (Table 3). Wilkinson et al. [18] employed investigator-based ratings of lens density during slit lamp examination in UM-HET3 mice. They reported an exacerbation of age-related lens density alterations under rapamycin treatment [18]. Assessing C57BL/6J Rj mice and using computer-assisted automated Scheimpflug imaging, Neff et al. found no clear effects of rapamycin on age-related changes in mean lens density [13]. Neff et al. [13] also used the virtual drum vision test to examine if rapamycin can prevent the aging-associate decline in visual acuity. While aged animals showed significantly reduced visual acuity, rapamycin treatment had no measurable effects on this aging trait. Together, the data available indicate that the lifespan-extending rapamycin treatment regimen pioneered by the ITP does not beneficially influence age-related ophthalmological changes and may even have adverse effects on specific aging traits of the eyes (i.e., changes in lens density).

**Table 3:** Ophthalmological findings

Assay	Aging phenotype	Intervention	Strain, sex	Treatment effects in aged mice	Treatment effects in young mice	References
Slit lamp examination with investigator-based rating of lens densities	Increased mean cataract score	Oral rapamycin (4.7, 14 or 42 ppm) initiated at 9 months of age	Male and female UM-HET3 mice	Cataract score further increased by treatment	Not examined	[18]
Scheimpflug imaging with computer-assisted automated measurements of lens densities	Increased mean lens density	Oral rapamycin (14 ppm) for approx. 1 year starting at 4 months or 13 months of age	Male C57BL/6J Rj	No effect	Not examined	[13]
Virtual drum vision test	Reduced visual acuity	Oral rapamycin (14 ppm) for approx. 1 year starting at 4 months or 13 months of age	Male C57BL/6J Rj	No effect	Not examined	[13]

## Cardiological findings

Aging is associated with structural and functional changes affecting the heart [66]. Echocardiography was employed in two studies to analyze the effects of the ITP rapamycin protocol on age-related structural and functional cardiac alterations [13, 19] (Table 4). In both studies, rapamycin was found to decrease a subset of heart dimensional measures, such as diastolic left ventricular internal diameter (LVIDd), and also decreased overall heart mass [13, 19]. Analyses in young animals demonstrated similar effects after a 3 months rapamycin treatment starting at 12 weeks of age [13], indicating age-independent drug effects on heart weight and heart dimensional measures.

**Table 4:** Cardiological findings

Assay	Aging phenotype	Intervention	Strain, sex	Treatment effects in aged mice	Treatment effects in young mice	References
Echocardiography	Changes in heart dimensions	Aging cohorts: oral rapamycin (14 ppm) for approx. 1 year starting at 4 months or 13 months of age; young animals: oral rapamycin (14 ppm) for 3 months starting at 12 weeks of age	Male C57BL/6J Rj	Reduced heart dimensions (LVIDd, LVIDs) and heart weight	Reduced heart dimensions (LVPWs) and heart weight	[13]
	Alterations in functional measures, such as cardiac output, ejection fraction, fractional shortening, as well as blood flow measurements and pressure gradients across heart valves			No measurable effects	Not examined	
Echocardiography	Changes in heart dimensions	Oral rapamycin (14 ppm) for 3 months started at 24 months of age	Female C57BL/6J	Reduced heart dimensions (LVSd, LVIDd, LVIDs, LVESV, LVEDV, LV mass) and heart weight	Not examined	[19]
	Functional measures: ejection fraction, fractional shortening; speckle-tracking strain analysis			Increases in these functional measures	Not examined	

Echocardiography also demonstrated the expected aging-associated decrease in functional cardiac measures, such as cardiac output, ejection fraction, fractional shortening, as well as blood flow measurements and pressure gradients across the aortic, pulmonary and mitral valves [13]. Neff et al. [13] assessed 26 months old male C57BL/6J Rj mice that had been treated for 13 months and found no significant rapamycin effects on any of these measures, suggesting limited effects of the drug on cardiac function. Flynn et al. [19] used a within-subjects design in female C57BL/6J mice, measuring cardiac function at 24 months of age and then again after 3 months of rapamycin or vehicle treatment. These authors report a small beneficial rapamycin effect on the ejection fraction and on fractional shortening [19]. Possible explanations for these different findings include differences in study design (between-subjects versus within-subjects), gender (male versus female) and treatment duration (with transient effects of treatment on these cardiac measures).

## Effects on the skeletal system and tendons

During the course of aging, there are typical changes affecting bones and the skeletal system, including decreases in trabecular bone volume and progressive kyphotic changes affecting the spine [19, 67]. The effect of a short-term rapamycin treatment (the ITP rapamycin protocol for 3 months, starting at 24 months of age) on spine kyphosis was examined using whole body micro CT [19] (Table 5). Measures were taken before commencement and after completion of the treatment. While a progressive change of spine kyphotic index was detected over this 3 months period, rapamycin had no measurable effects on the degenerative changes [19].

**Table 5:** Bones and skeletal system

Assay	Aging phenotype	Intervention	Strain, sex	Treatment effects in aged mice	Treatment effects in young mice	Reference
Whole body micro CT	Increasing kyphosis of the spine	Oral rapamycin (14 ppm) for 3 months started at 24 months of age	Female C57BL/6J	No measurable effects	Not examined	[19]
Micro CT analysis of tibiae	Decrease in trabecular bone volume	Hypomorphic *mTOR* allele (*mTOR* ^∆/∆^);* mTOR* ^∆/∆^ mice were compared to wildtype littermate controls; young animals were 3–6 months old; aged animals were 17–27 months old	Mixed 129S1 and C57BL/6Ncr background; female groups of mice	Further decrease of trabecular bone volume in aged *mTOR* ^∆/∆^ mice (caveat: small sample size with group sizes of 4–6 animals)	No detectable genotype difference in young mice	[17]
Mechanical tendon evaluation (tibialis anterior tendon), assessment at 22 months of age	Increase in maximum tangent modulus (a measure indicating resistance to stretching); decrease in hysteresis (a measure indicating the extent to which tendons recover to their original length in the unstretched condition)	Oral rapamycin (14 ppm) initiated at 9 months of age	Female UM-HET3 mice	Decreased maximum tangent modulus and increased hysteresis in aged mice	Not examined	[18]

Micro CT analyzes were also employed to measure tibial bone density in young and aging *mTOR* ^∆/∆^ mice, as well as corresponding wildtype controls [17] (Table 5). These analyzes showed the expected loss of trabecular bone volume in the tibiae of aged mice. The *mTOR* ^∆/∆^ genotype had no beneficial effects on age-related loss of bone volume, but instead appeared to lead to a further exacerbation of this aging phenotype [17]. No difference in trabecular bone volume was detected between young *mTOR* ^∆/∆^ mice and age-matched wildtype controls [17]. Together, the data available [17, 19] suggest that pharmacological and genetic mTOR inhibition may have limited effects on specific age-related skeletal and bone changes.

Wilkinson et al. [18] examined age-related changes in the mechanical properties of tibialis anterior tendons (Table 5). Aged mice showed a significant increase in maximum tangent modulus (a measure indicating resistance to stretching) and a decrease in hysteresis (a measure indicating the extent to which tendons recover to their original length in the unstretched condition) [18]. Rapamycin treatment (the ITP protocol) significantly improved these age-related biomechanical tendon properties [18]. Rapamycin effects on tendons in young animals were not included in the analyses. Future studies have to address if rapamycin has symptomatic effects on tendon properties in aged mice or if it slows the age-related development of these alterations.

## Clinical chemistry, hematology and immunology

Murine aging is associated with an altered cellular composition of the peripheral blood [68, 69]. Blood cell counts were measured in three aging mouse cohorts that were treated with rapamycin or vehicle control for approx. 1 year (starting at either 4, 13 or 25 months) before analyses commenced [13] (Table 6). Aged animals showed elevated white blood cell counts and elevated platelet counts, both of which were not modified by rapamycin treatment [13]. Additionally, old animals showed reduced red blood cells counts associated with decreased mean corpuscular volume (MCV), mean corpuscular hemoglobin (MCH) and increased red blood cell distribution width (RDW) [13]. Together, these findings are reminiscent to hematology findings associated with iron deficiency anemia, anemia of chronic diseases and anemia caused by chronic bleeding. Rapamycin treatment significantly elevated red blood cell counts in aged animals and tended to have similar effects on red blood cell counts in young animals ([13]; see also [70]), indicating that rapamycin effects were likely linked to aging-independent effects on erythrocyte production and/or turnover.

**Table 6:** Clinical chemistry, hematology and immunology

Assay	Aging phenotype	Intervention	Strain, sex	Treatment effects in aged mice	Treatment effects in young mice	Reference
Blood cell counts	Reduced red blood cell couns, increased white blood cell counts and platelets	Aging cohorts: oral rapamycin (14 ppm) for approx. 1 year starting at 4 months, 13 months or 25 months; young animals: oral rapamycin (14 ppm) for 3 months starting at 12 weeks of age	Male C57BL/6J Rj	Rapamycin increased red blood cell counts; no effect on white blood cell counts and platelets	Increase of red blood cell counts	[13]
Clinical chemistry	Increased plasma sodium, calcium, chloride, total protein, albumin, alkaline phosphatase, and α-amylase; decreased triglycerides	Oral rapamycin (14 ppm) for approx. 1 year starting at 4, 13 or 25 months	Male C57BL/6J Rj	No amelioration of these aging traits	Not examined	[13]
Immunology (FACS)	Increased CD25+CD4+ T cell population; decreased γδ T cell population; increased CD44^hi^ T cell populations	Aging cohorts: oral rapamycin (14 ppm) for approx. 1 year starting at 4, 13 or 25 months; young animals: oral rapamycin (14 ppm) for 3 months starting at 12 weeks of age	Male C57BL/6J Rj	Decreased CD25+ CD4+ T cell population; increased γδ T cell population; decreased CD44^hi^ T cell populations	Decreased CD25+CD4+ T cell population; no effect on γδ T cell and CD44^hi^ T cell populations (but see [48] and [56])	[13]
	Decreased CD4+ T lymphocytes; decreased NK cells and NK/CD11b+ cells; increased IgD^hi^ B cells and MHCII^hi^ B cells			No measurable amelioration of aging traits	Not examined	
Immunoglobulin measurements	Increased plasma immunoglobulin concentrations			Tended to decrease plasma immunoglobulins (in part)	Not examined	

Detailed clinical chemistry assessments showed clear aging-associated changes in plasma concentrations of sodium, calcium, and chloride (increased), glucose (decreased), and triglycerides (decreased) [13], which is in agreement with previously reported murine aging traits [68]. Additionally, old mice showed increased alkaline phosphatase and α-amylase levels in their plasma [13]. Rapamycin treatment had by and large no detectable effects on these clinical chemistry aging traits (except for an elevation of blood glucose) [13].

Ten-color polychromatic flow cytometry was used to examine quantitative aging and/or rapamycin effects on various immune cell populations in the three aging mouse cohorts mentioned above [13]. Aged animals showed the expected strong reduction in the frequency of CD4+ T cells, an increase in the frequency of CD44^hi^ T cells (indicative of an activated/memory T cell phenotype) and a strong reduction in the frequency of NK cells, among additional alterations [13]. Rapamycin treatment had no measurable effects on the frequency of CD4+ T cells and NK cells, but counteracted the age-related change in CD44^hi^ T cells [13]. Rapamycin did not influence the frequency of CD44^hi^ T cells in the young group of animals examined in the Neff et al. [13] study. Nevertheless, it still remains unclear if rapamycin specifically has this effect on CD44^hi^ T cells in aged animals because genetic mTOR inhibition has been shown previously to lead to reduced CD44^hi^ T cell counts in young animals [71].

Immunoglobulin plasma concentrations are often robustly elevated in aged mice [13]. Rapamycin tended to decrease plasma immunoglobulin concentrations in several cases [13]. These analyses were only carried out in the aging cohorts and it remains to be determined if a more acute rapamycin treatment may have similar consequences in young mice.

## Metabolism

Indirect calorimetry was used to assess metabolic changes in aged animals and their possible modulation by rapamycin [13] (Table 7). These studies showed that certain parameters seen in aged mice, such as reduced oxygen consumption and lower body temperature, were not restored by rapamycin treatment [13]. Rapamycin had an effect on the respiratory exchange ratio, at least in one of the examined cohorts [13], that deserves further attention in future studies.

**Table 7:** Metabolism

Assay	Aging phenotype	Intervention	Strain, sex	Treatment effects in aged mice	Treatment effects in young mice	Reference
Indirect calorimetry	Reduced oxygen consumption; lower body temperature	Aging cohorts: oral rapamycin (14 ppm) for approx. 1 year starting at 4, 13 or 25 months; young animals: oral rapamycin (14 ppm) for 3 months starting at 12 weeks of age	Male C57BL/6J Rj	No measurable effect	Not examined	[13]
	Reduced respiratory exchange ratio (RER)			Increased RER	No measurable effect	

## Pathology findings

A number of experiments have been carried out to study rapamycin’s effects on histopathological aging phenotypes in mice. The data available to date are described below and are summarized in Table 8.

**Table 8:** Pathology

Assay	Aging phenotype	Intervention	Strain, sex	Treatment effects in aged mice	Treatment effects in young mice	References
*Brain*						
Histopathological assessment of adult hippocampal neurogenesis	Reduced doublecortin expression in the dentate gyrus	Oral rapamycin (14 ppm) for approx. 1 year starting at 4 months	Male C57BL/6J Rj	No effect	Not examined	[13]
Immunostainings for polyubiquitinated proteins and nitrotyrosine in brain sections	Increased polyubiquitinated proteins; increased intensity of nitrotyrosine staining	Hypomorphic *mTOR* allele (*mTOR* ^∆/∆^); *mTOR* ^∆/∆^ mice were compared to wildtype littermate controls; young animals were 3–6 months old; aged animals were 17–27 months old	Mixed 129S1 and C57BL/6Ncr background; female groups of mice	Decreased polyubiquitinated proteins and nitrotyrosine staining intensity	Not examined	[17]
*Skeletal muscle*						
Histopathological assessment of cross-sectional muscle fiber area	Reduced cross-sectional muscle fiber area	Oral rapamycin (14 ppm) for approx. 1 year starting at 4 or 13 months of age	Male C57BL/6J Rj	No effect	Not examined	[13]
*Heart and aorta*						
Histopathology, heart	Myocardial pathology, such as ventricular dilation, myocardial hypertrophy, fibrosis and thickening of the heart valves	Oral rapamycin (14 ppm) for approx. 1 year starting at 4, 13 or 25 months of age	Male C57BL/6J Rj	No measurable effects	Not examined	[13]
Histopathology, nuclei of cardiac myocytes	Abnormalities of nuclear size and chromatin conformation	Oral rapamycin (4.7, 14 or 42 ppm) initiated at 9 months of age	Male and female UM-HET3 mice	Reduced frequency of atypical nuclei (caveat: finding borderline significant using a one-sided statistical analysis)	Not examined	[18]
Histopathology, aorta	Arterial degeneration (deposition of mucinous substance, elastic fiber fragmentation)	Oral rapamycin (14 ppm) for approx. 1 year starting at 4, 13 or 25 months of age	Male C57BL/6J Rj	No measurable effects (caveat: limited number of observations)	Not examined	[13]
*Liver*						
Histopathology, liver	Periportal fibrosis, polyploidy; liver steatosis	Oral rapamycin (14 ppm) for approx. 1 year starting at 4, 13 or 25 months of age	Male C57BL/6J Rj	No measurable effects	Not examined	[13]
	Microgranulomas			Decreased prevalence of microgranulomas		
Histopathology, liver	Multifocal macrovesicular lipidosis	Oral rapamycin (4.7, 14 or 42 ppm) initiated at 9 months of age	Male UM-HET3 mice	Reducing the proportion of animals affected	Not examined	[18]
*Kidney*						
Histopathology, kidney	Glomerulosclerosis	Oral rapamycin (14 ppm) for approx. 1 year starting at 4, 13 or 25 months of age	Male C57BL/6J Rj	No detectable effects	Not examined	[13]
	Vacuolization of tubulus epithelia cells; hyperplasia of tubulus epithelia cells			Treatment exacerbated vacuolization of tubulus epithelia cells (toxic tubulus damage)		
*Adrenal glands*						
Histopathology, adrenal glands	Lipofuscin deposition	Oral rapamycin (14 ppm) for approx. 1 year starting at 4, 13 or 25 months	Male C57BL/6J Rj	No measurable effects	Not examined	[13]
*Thyroid gland*						
Histopathology, thyroid gland	Increased thyroid follicle size	Aging cohorts: oral rapamycin (14 ppm) for approx. 1 year starting at 4, 13 or 25 months; young animals: oral rapamycin (14 ppm) for 3 months starting at 12 weeks of age	Male C57BL/6J Rj	Decreased thyroid follicle size	Decreased thyroid follicle size	[13]
*Male reproductive tract*						
Histopathology, male reproductive tract	Testis atrophy	Oral rapamycin (4.7, 14 or 42 ppm) initiated at 9 months of age	Male UM-HET3 mice	Testicular degeneration	Not examined	[18]
Histopathology, male reproductive tract	Testis atrophy	Oral rapamycin (14 ppm) for approx. 1 year starting at 4, 13 or 25 months	Male C57BL/6J Rj	Testicular degeneration	Not examined	[13]
*Female reproductive tract*						
Histopathology, female reproductive tract	Endometrial hyperplasia	Oral rapamycin (4.7, 14 or 42 ppm) initiated at 9 months of age	Female UM-HET3 mice	Reduced frequency of endometrial hyperplasia (caveat: finding borderline significant and only when comparing untreated animals against one of the dosing groups)	Not examined in this study; findings in animals prone to endometrial hyperplasia, however, show similar treatment effect in young animals [76]	[18]

One of the well-documented age-related alterations is the strong decline in adult hippocampal neurogenesis [72]. Neff et al. [13] determined if a 1 year rapamycin treatment (the ITP protocol), initiated at 4 months, prevents the age-related decline in hippocampal neurogenesis. They observed the expected clear reduction in doublecortin immunoreactivity in the dentate gyrus, which was, however, not significantly modulated by rapamycin [13], indicating that the treatment had no measurable effects on age-related alterations in adult hippocampal neurogenesis.

There is an accumulation of polyubiquitinated and nitrotyrosinylated proteins in the aging mouse brain. Wu et al. [17] employed immunohistochemical analyses of brain sections to measure possible effects of a hypomorphic *mTOR* mutation on these aging traits. Their data showed a reduced immunoreactivity for polyubiquitinated and nitrotyrosinylated proteins in brain sections of aged mTOR mutants compared to age-matched wildtype controls [17], suggesting that the appearance of these aging traits was suppressed in the mutants.

The aging-associated loss in muscle strength (discussed above) is paralleled by an atrophy of skeletal muscle fibers (sarcopenia) [73]. Neff et al. [13] quantitatively analyzed cross-sectional muscle fiber surface area of the quadriceps femoris muscle of aged animals treated either with rapamycin or vehicle control. Aged animals showed the expected reduction in cross-sectional muscle fiber surface area and rapamycin did not ameliorate this aging phenotype [13].

Aging of the cardiovascular system is associated with a broad set of histopathological alterations. Neff et al. [13] performed detailed histopathological analyses of aging-associated myocardial changes (such as ventricular dilation, myocardial hypertrophy, fibrosis and thickening of the heart valves) and degenerative arterial alterations, which showed no obvious preventative effects of rapamycin treatment (the ITP protocol) on these aging traits. Wilkinson et al. [18] assessed rapamycin’s effects on morphological changes affecting cardiomyocytes in the aged heart (abnormalities in nuclear size and chromatin conformation): Their findings suggest that rapamycin may have reduced the frequency of this alteration in aged animals, which should encourage future studies to re-examine this rapamycin effect. It was not assessed if rapamycin has effects on nuclear size and chromatin conformation of cardiomyocytes in young animals. It is therefore currently unknown if this finding is better explained by a general age-independent drug effect or by specific prevention of a cardiac aging phenotype.

Age-related liver phenotypes were examined histopathologically in the context of the Neff et al. and the Wilkinson et al. study. Neff et al. [13] observed no preventative rapamycin effect on periportal fibrosis and polyploidy of hepatic cells, which are both substantially more common in aged animals than in young controls. Rapamycin did, however, decrease the frequency of microgranulomas in the liver of animals aged on the drug [13]. Wilkinson et al. [18] examined rapamycin’s effects on multifocal macrovesicular lipidosis of the liver, which is common in aged male, but not female UM-HET3 mice. Rapamycin treatment seemed to decrease the prevalence of this trait in aged male UM-HET3 mice in a dose-dependent fashion [18]. It is currently unclear if this rapamycin effect is reflective of a prevention of aspects of liver aging or if it is related to more immediate drug effects on hepatic lipid metabolism [74].

Renal age-related changes include glomerulosclerosis and alterations affecting the renal tubular system. Neff et al. [13] assessed rapamycin effects on the prevalence of glomerulosclerosis in aged mice and on histopathological changes affecting tubulus epithelia cells (vacuolization of tubulus epithelia; hyperplasia of tubulus epithelia). There was no detectable beneficial effect on glomerulosclerosis [13]. Rapamycin treatment, however, resulted in a significantly increased prevalence of vacuolization of tubulus epithelial cells [13], indicative of nephrotoxic side effects of rapamycin treatment.

In the thyroid gland, aging is associated with increased follicle sizes and decreased colloid resorption [13, 75]. Neff et al. [13] examined thyroid follicle size distributions in mice aged on rapamycin (oral, 14 ppm) or vehicle control diet. The data showed the expected substantial age-related increase in thyroid follicle sizes [13]. Rapamycin treatment significantly decreased thyroid follicle size in aged mice [13]. However, the drug also led to a reduction in thyroid follicle size in young animals (in which a 3 months treatment was initiated at 12 weeks of age) [13], indicating that rapamycin effects on thyroid follicle size in aged animals cannot be attributed to a slowing of aging. In the adrenal glands, aging is associated with a prominent deposition of lipofuscin within the adrenal gland parenchyma. Rapamycin treatment did not ameliorate lipofuscin deposition in the adrenal glands [13].

Rapamycin effects on the male reproductive tract were examined histopathologically in two studies published to date [13, 18]. Wilkinson et al. treated UM-HET3 mice with three different doses of oral rapamycin (4.7, 14, 42 ppm), starting at 9 months of age and assessing the animals at 22 months [18]. Neff et al. [13] assessed C57BL/6J mice that were treated with 14 ppm of oral rapamycin for approx. 1 year (starting at 4, 13 and 20–22 months, respectively) before assessment commenced. In both studies significant testicular degeneration was observed in rapamycin-treated animals, indicating that this is a robust side effect of rapamycin that is seen at various doses and across different genetic backgrounds.

Aging-associated changes in the uterus include an increased prevalence of endometrial hyperplasia. Wilkinson et al. assessed rapamycin effects on the frequency of endometrial hyperplasia in aged female UM-HET3 mice [18]. The authors report a significant decrease in the prevalence of endometrial hyperplasia when comparing the rapamycin group with the highest dose (42 ppm orally) against the untreated aged controls [18]. It is currently unclear if this rapamycin effect reflects a slower rate of aging under treatment. More immediate, aging-independent rapamycin effects could well-explain the inhibition of endometrial hyperplasia given that inactivating *Tsc2* mutations (an important mTOR repressor) were shown to result in endometrial hyperplasia, which could then be suppressed by pharmacological mTOR inhibition [76].

## Gene expression

Using the ITP rapamycin adminstration protocol [10], two complementary studies investigating rapamycin-induced gene expression changes in the liver of aged mice were published recently [77, 78].

The first one performed transcriptome analysis of liver tissues derived from 25 months old C57BL/6 mice which were fed with 14 ppm encapsulated rapamycin starting at 4 months (for 21 months) or 19 months (for 6 months), respectively [77]. Despite of considerably extended lifespan in both genders, changes of gene expression induced by long term rapamycin treatment were apparently greater in females (2,504 genes up-regulated and 2,257 down-regulated) than males (159 genes up-regulated and 129 down-regulated) [77] (Table 9). Results obtained from chronically rapamycin fed males, however, remained ambiguous because of profound inner gender heterogenity: approximately, half of the animals (7 of 13) showed a profile similar to control fed males, while the other half (6 of 13) exhibited general similarity to rapamycin fed females. One possibility is that effects in females were more consistent because of higher rapamycin blood levels at a given dose [15]. Shorter-term rapamycin treatment (for 6 months) yielded expression alterations of 100 genes (32 up-regulated and 68 down-regulated) in males and 1,427 genes (675 up-regulated and 752 down-regulated) in females (Table 9). Using ingenuity pathway analysis (IPA), genes significantly affected by chronic rapamycin treatment were categorized into 13 different pathways. Two of those pathways (mitochondrial function and protein degradation) were also modulated by the 6 months treatment (Table 9).

**Table 9:** Rapamycin and gene expression studies using microarrays in mice

Strain, sex	Intervention	Organ	Genes up-regulated	Genes down-regulated	Pathways involved	Reference
Male and female C57BL/6J	Oral rapamycin (encapsulated, 14 ppm) initiated at 4 months of age for 21 months	Liver	159 (males), 2,504 (females)	129 (males), 2,257 (females)	13	[77]
	Oral rapamycin (encapsulated, 14 ppm) initiated at 19 months of age for 6 months		32 (males), 675 (females)	68 (males), 752 (females)	3	
Male C57BL/6	Oral rapamycin (encapsulated, 14 ppm) initiated at 2 months of age with duration of 6 months	Liver	783	628	105	[78]
	40 % dietary restriction initiated at 2 months of age with duration of 6 months		1,621	256	88	
	40 % dietary restriction combined with oral rapamycin treatment (encapsulated, 14 ppm) initiated at 2 months of age with duration of 6 months		2,558	1,130	170	

The second study aimed to compare liver transcriptome profiles of rapamycin fed male mice and those under a 40 % dietary restriction (DR) regimen starting at 2 months until 8 months of age [78]. The vast majority of gene expression modifications were restricted either to DR or rapamycin treatment as there was only an overlap of a small subset of genes (490 up-regulated genes (=26 %) and 74 down-regulated genes (=9 %) detected. Moreover, according to IPA analysis, ubiquitination represented the only top ranked pathway in both DR and rapamycin treated groups. Interestingly, DR combined with rapamycin led to additional gene expression changes (1,049 genes up-regulated and 767 down-regulated) not found by DR or rapamycin treatment alone (Table 9).

In summary, both studies provide initial insights of rapamycin associated gene regulation in a single organ (the liver). Unfortunately, the question whether those changes are directly linked with aging phenomena remained unanswered since the first report did not include young mice and the second one was limited to young male animals. In the future, more comprehensive transcriptome analyses including multiple tissue types and organs are required to fully elucidate the connection between rapamycin regulated gene expression and aging.

## Conclusions and future directions

Rapamycin effects on numerous aging traits have been analyzed to date (as outlined above), mostly employing the original rapamycin administration regimen developed by the ITP that was shown to extend lifespan in mice [10]. Although there were a considerable number of aging traits not modified by treatment, rapamycin afforded improvement of a subset of traits examined [11–14, 18–22]. The aging traits found to be ameliorated by rapamycin were either related to immune system changes (e.g., plasma immunoglobulin concentrations, frequency of specific T cell subsets, cytokine concentrations in blood and heart, response to vaccination), age-related alterations in body mass, organ size and dimensions (body weight, fat mass, lean mass, thyroid follicle size, cardiac dimension, heart weight), tumors and pre-cancerous lesions, as well as neurobehavioral changes (motor activity, learning and memory). Where available [13], the data indicate that similar effects are seen using shorter-term treatments in young adult animals, indicative of aging-independent drug effects. More work is, therefore, needed to determine to what extend these rapamycin effects on the aged organism reflect symptomatic improvements (such as seen on a number of traits in the Neff et al. [13] study) versus a true slowing of aging processes. Although many drug effects are seen with the ITP rapamycin protocol, it will also be important to assess higher doses that may allow for a more complete mTOR inhibition in target tissues.
